# Enhanced thermoelectric properties in Sb/Ge core/shell nanowires through vacancy modulation

**DOI:** 10.1038/s41598-021-01301-7

**Published:** 2021-11-09

**Authors:** Prabal Dev Bhuyan, P. N. Gajjar, Rita Magri, Sanjeev K. Gupta

**Affiliations:** 1 Computational Materials and Nanoscience Group, Department of Physics and Electronics, St. Xavier’s College, Ahmedabad, 380009 India; 2 Department of Physics, Gujarat University, Ahmedabad, 380009 India; 3 Department of Physics, Informatics and Mathematics (FIM), University of Modena and Reggio Emilia, Via Campi 213/A, Modena, Italy

**Keywords:** Electronic properties and materials, Nanowires

## Abstract

In the present work, we have modified the physical and electronic structure of Sb/Ge core/shell nanowires via vacancy creation and doping with foreign atoms with the aim to improve their thermoelectric energy conversion efficiency. Sb/Ge-NWs having a diameter of 1.5 Å show metallicity with 2G_o_ quantum conductance. The stability of the nanowires is assessed through the calculation of their formation energy. The formation of one vacancy at either the Sb- and Ge-site modifies substantially the electronic properties. From the comparison of the thermoelectric properties of the nanowires with and without the vacancy, we have found that the figure of merit for the Sb/Ge NW with one Sb vacancy increases of 0.18 compared to the pristine NW. The NW doping with different transition metals: Fe, Co, Ni and Cu have been found to also enhance the conversion efficiency. Thus, our calculations show that the thermoelectric performance of metal–semiconductor core–shell NWs can be in principle improved as much as 80% by vacancy formation and doping.

## Introduction

Thermoelectric materials have attracted a huge scientific interest as sustainable energy resources due to their ability to convert waste heat into electricity^1–3^. These thermoelectric material devices can transform waste heat given off from sources such as power plants, motor vehicles, computers or human bodies into electric power^4–10^. The conversion of thermal energy due to a gradient of temperature into electrical energy is known as Seebeck effect and the reverse counterpart of this phenomenon is the Peltier effect^11,12^. The thermo-electric energy conversion is also an essential requirement for nano-electronic, optoelectronic and photonic devices that need removal of the unwanted produced heat^13–16^. The conversion efficiency of these materials is quantified by calculating the thermoelectric figure of merit (ZT)^4^ defined as;

1 $$ ZT = \frac{{S^{2} \sigma T}}{\kappa } $$

where, S is the Seebeck coefficient, σ is the electrical conductivity and T is the temperature. $\kappa$ is the thermal conductivity, defined by $\kappa = \kappa_{e} + \kappa_{p}$; where $\kappa_{e}$ and $\kappa_{p}$ are the electronic and phononic thermal conductivity, respectively. From Eq. (1), it is clear that a high performance thermoelectric material should possess a high power factor ($PF = S^{2} \sigma$) and a low thermal conductivity.

It is always a big challenge to enhance the thermoelectric efficiency of a material. Metals have a higher electrical conductivity, but their Seebeck co-efficient is low due to the symmetry of their density of states across the Fermi energy. Such feature leads to low ZT values^17^, since the number of hot electrons above the Fermi energy is roughly the same as the number of cold empty states under the Fermi energy. Therefore, under a temperature gradient, the number of electrons diffusing from the hot side to the cold side is approximately equal to the number of electrons diffusing from the cold side to the hot side leading to a low Seebeck co-efficient. This effect is not observed in semiconductors due to the presence of a band gap, which allows only one type of charge carrier to diffuse^17^. Recently, it was reported that the reduction of dimensionality is one of the most promising methods to enhance the conversion efficiency^1,18–22^. In bulk structures, phonons are the main heat carriers, responsible of a generally high thermal conductivity. However, as a consequence of the reduction of dimensionality, the phonon boundary scattering increases and the phononic contribution to the thermal conductivity decreases^16,19,23–26^. The nanostructuring of a material also increases the Seebeck coefficient due to the presence of sharp features in the electronic density of states near the Fermi level^27^. In this regard, Shafique et al. have reported that two-dimensional (2D) monochalcogenides have a better thermoelectric performance than their bulk phases. They reported high values of the figure of merit (> 1) for SnSe, SnS, GeSe and GeS monolayers^18^. It is also reported that although bulk silicon (Si) is a poor thermoelectric material, Si nanowires show promise as high performance thermoelectric materials because of a reduced thermal conductivity^15,28–33^. Recently, Peng et al. have compared the electronic and thermoelectric properties of SbSeI, SbSI and SbSBr NWs with those of their bulks and observed that 1D SbSeI has a much larger Seebeck co-efficient than bulk SbSeI, indicating a higher thermoelectric performance^27^. Further, Kim et al. have fabricated Bi/Te core/shell nanowires with various diameters and investigated their thermoelectric properties. They have reported that the electrical conductivity and Seebeck co-efficient increase with increasing the nanowire diameter until they maximize at diameters exceeding 400 nm. They have observed a maximum thermoelectric figure of merit of 0.5 for a Bi/Te NW (456 nm diameter) at room temperature, which is greater than the reported values for pure Bi NWs (0.07) and bulk Bi (0.05)^34^. These results motivated us to study the thermoelectric properties of core/shell nanowires.

In this work, we have considered Sb/Ge core/shell nanowires. GeSb-NWs of diameter 40–100 nm have been grown experimentally and shown to have application in memory devices^35^. We have studied the electronic and thermoelectric properties of model Sb/Ge NWs also investigating how these properties change with the presence of one vacancy in either the core or shell regions. Further, we have also modelled the doping of the NWs by adding Fe, Co, Ni and Cu transition metal atoms to the NW shell to study if and how the energy conversion efficiency is affected. We have calculated the thermal conductivity, the Seebeck co-efficient and the electronic figure of merit (ZT_e_) for each structural modification of the NWs.

## Computational details

We have employed the density functional theory (DFT) as implemented in the SIESTA simulation package to optimize the atomic structure^36^. The generalized gradient approximation (GGA) with the Perdew-Burke-Ernzerhof parameterization (PBE) is used to treat the exchange and correlation energies^37^. The energy minimization is performed by employing a standard conjugate-gradient (CG) technique and a split-valence double zeta basis set with polarization function (DZP)^38^. The nanowires are relaxed until the forces on each atom are less than 0.01 eV/Å. We use a 450 Ry energy cutoff and the Brillouin zone is sampled with a 1 × 1 × 12 Monkhorst Pack grid. A vacuum region of about 20 Å along the x and y directions is used to prevent interactions between periodic images.

From the converged DFT calculations, the underlying mean-field Hamiltonian, combined with the tool GOLLUM, which is based on equilibrium transport theory, is used to study the thermoelectric properties^39^. Thermoelectric properties such as the electrical conductance G(T), the electronic contribution to the thermal conductance κ_e_ (T), the thermo-power S(T) and the Peltier co-efficient $\Pi (T)$ of the material as functions of the temperature are given by^19^,

2 $$ G(T) = G_{0} L_{0} $$

3 $$ \kappa_{e} (T) = \frac{{L_{0} L_{2} - L_{1}^{2} }}{{hTL_{0} }} $$

4 $$ S(T) = - \frac{{L_{1} }}{{eTL_{0} }} $$

5 $$ \Pi (T) = TS(T) $$

where

6 $$ L_{n} (T) = \int\limits_{ - \infty }^{ + \infty } {dE(E - E_{F} )^{n} T(E)\left( { - \frac{df(E)}{{dE}}} \right)} $$

$$T(E)$$ is the transmission co-efficient, $$f(E)$$ the Fermi–Dirac probability distribution function, *T* the temperature and E_F_ the Fermi energy. G_0_ is the quantum of conductance, $$G_{0} = {\raise0.7ex\hbox{${2e^{2} }$} \!\mathord{\left/ {\vphantom {{2e^{2} } h}}\right.\kern-\nulldelimiterspace} \!\lower0.7ex\hbox{$h$}}$$. From the values of the Seebeck coefficient, conductance, and thermal conductivity, we calculate the electronic contribution to the figure of merit,

7 $$ ZT_{e} = \frac{{L_{1}^{2} }}{{L_{0} L_{2} - L_{1}^{2} }} $$

## Results and discussion

### Electronic properties

**Figure 1:**
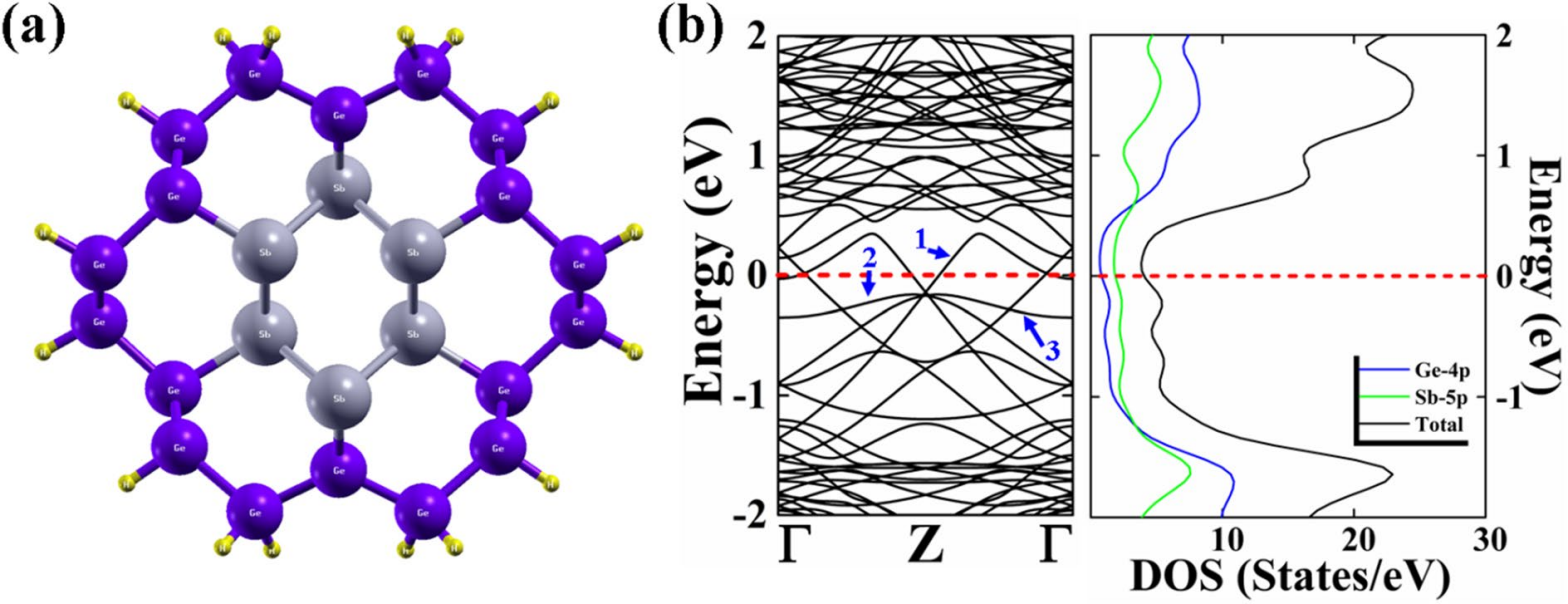
(**a**) Optimized electronic structure of the Sb/Ge core/shell nanowire. The electronic band structure and partial density of states of the NW are shown in (**b**). The Fermi energy is set at 0 eV. Grey balls denote Sb atoms; violet balls denote Ge atoms and the yellow balls the hydrogen atoms.

We have considered a Sb/Ge core/shell nanowire, in which a core of antimony (Sb) is wrapped with a shell of one monolayer of germanium (Ge) as shown in Fig. 1a. There are 40 atoms in the unit cell. The core region consists of 6 atoms of Sb and the shell region consists of 18 atoms of Ge. The NW is passivated with 16 hydrogen atoms to prevent the dangling bond states to fall in the energy region of interest for the calculation of the electronic properties. The diameter of the NW is 1.5 Å.

We have calculated the energy of formation (FE) of the core–shell structure in the Sb/Ge nanowire to assess the thermodynamic stability. The FE is calculated under zero pressure and by neglecting the vibrational and the zero-point energy contributions. We have used the following formula

8 $$ FE = E_{tot} - E_{tot} (GeH) + nE_{bulk} (Ge) - nE_{bulk} (Sb) $$

where, $E_{tot}$ is the core–shell nanowire total energy, $E_{tot} (GeH)$ is the total energy of the hydrogen passivated NW with all Ge atoms. $E_{bulk} (Ge)$ is the energy of one Ge atom in its bulk and $E_{bulk} (Sb)$ is the optimized energy of one atom of Sb in the same bulk structure as in the NW. $n$ is the number of Ge atoms substituted in the core with Sb atoms. The FE for the core/shell nanowire is calculated to be negative, − 0.0225 eV. Our calculated FE accounts for the energy associated to the creation of the mixed Ge–Sb bonds and for the following structural relaxation of the entire NW. The negative value is associated to the efficient atomic relaxation occurring in the thin NWs and assures that, once the NWs are synthesized (the NW synthesis is dominated by kinetic factors not by thermodynamics) the NWs can maintain their core–shell structure at low temperatures.

We studied the electronic band structure of the NWs as shown in Fig. 1b. Band lines are crossing the Fermi energy and the NWs show a metallic behaviour. The electronic properties of the Sb/Ge NWs are different from those of the pure Sb and Ge-NWs. The change in the electronic properties is due to the induced compressive strain experienced by the metal core region due to its lattice-mismatch with the shell region as we have explained in our previous work^40^. The band lines crossing the Fermi energy give rise to a quantum conductance measured in units of G_0_ = 2e^2^/h, where e is the unit of charge and h is the Planck’s constant^41^. This conductance gives information about the electron transport in the NW in the absence of electron–electron and electron–phonon scattering. The quantum conductance of the Sb/Ge-NW is calculated to be 2G_0_.

In order to get a further insight into the electronic properties, we have calculated the partial densities of states (PDOS) to study the orbital contributions of the atoms near the Fermi level (Fig. 1b). Contributions of both Sb-5*p* and Ge-4*p* orbitals are observed near the Fermi energy. The Sb-5*p* orbital contribution is larger at the Fermi energy than that of the 4p orbital of Ge, while the Ge-4*p* orbital has a larger density of states far away from the Fermi level into the conduction and valence bands.

**Figure 2:**
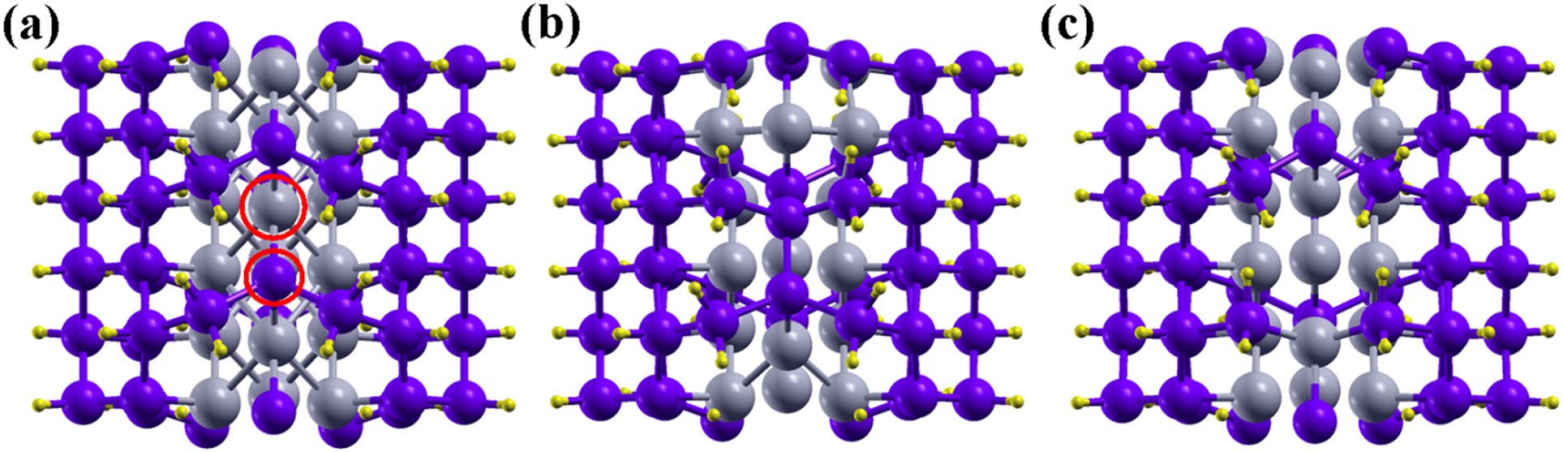
(**a**) (1 × 1 × 3) supercell of pristine Sb/Ge NWs where the red circles indicate the sites of the Ge and Sb vacancies (**b**) optimized structure of the Sb/Ge NWs with Sb vacancy and (**c**) optimized structure of the Sb/Ge NWs with Ge vacancy.

Next, we have studied the electronic properties of the NWs containing one Sb (Ge) vacancy. We have optimized the (1 × 1 × 3) supercell structure of defective Sb/Ge NWs as shown in Fig. 2. Figure 2a shows the chosen vacancy sites. The removed Sb atom is one in the core while the removed Ge atom is one in the shell bonded to Sb atoms. The optimized structure of Sb/Ge NWs with Sb and Ge vacancy are shown in Fig. 2b and c, respectively. The bond lengths and bond angles are slightly distorted near the vacancy site. The distortion is larger in the case of the Sb core vacancy.

The vacancy formation energy is given as^42^:

9 $$ E_{f} = E_{vacancy} - E_{pristine} - n_{i} \mu_{i} $$

where, $E_{vacancy}$ and $E_{pristine}$ are the total energies with and without the vacancy, respectively. $\mu_{i}$ is the chemical potential of *i*th atomic species and $n_{i}$ is the number of removed atoms. The chemical potential is the energy of one Sb (Ge) atom in its bulk. The Ge vacancy formation energy is calculated to be − 10.59 eV and the Sb vacancy formation energy is − 8.84 eV. The negative formation energy of the Sb and Ge vacancies in the Sb/Ge NW indicates that they are energetically favorable.

**Figure 3:**
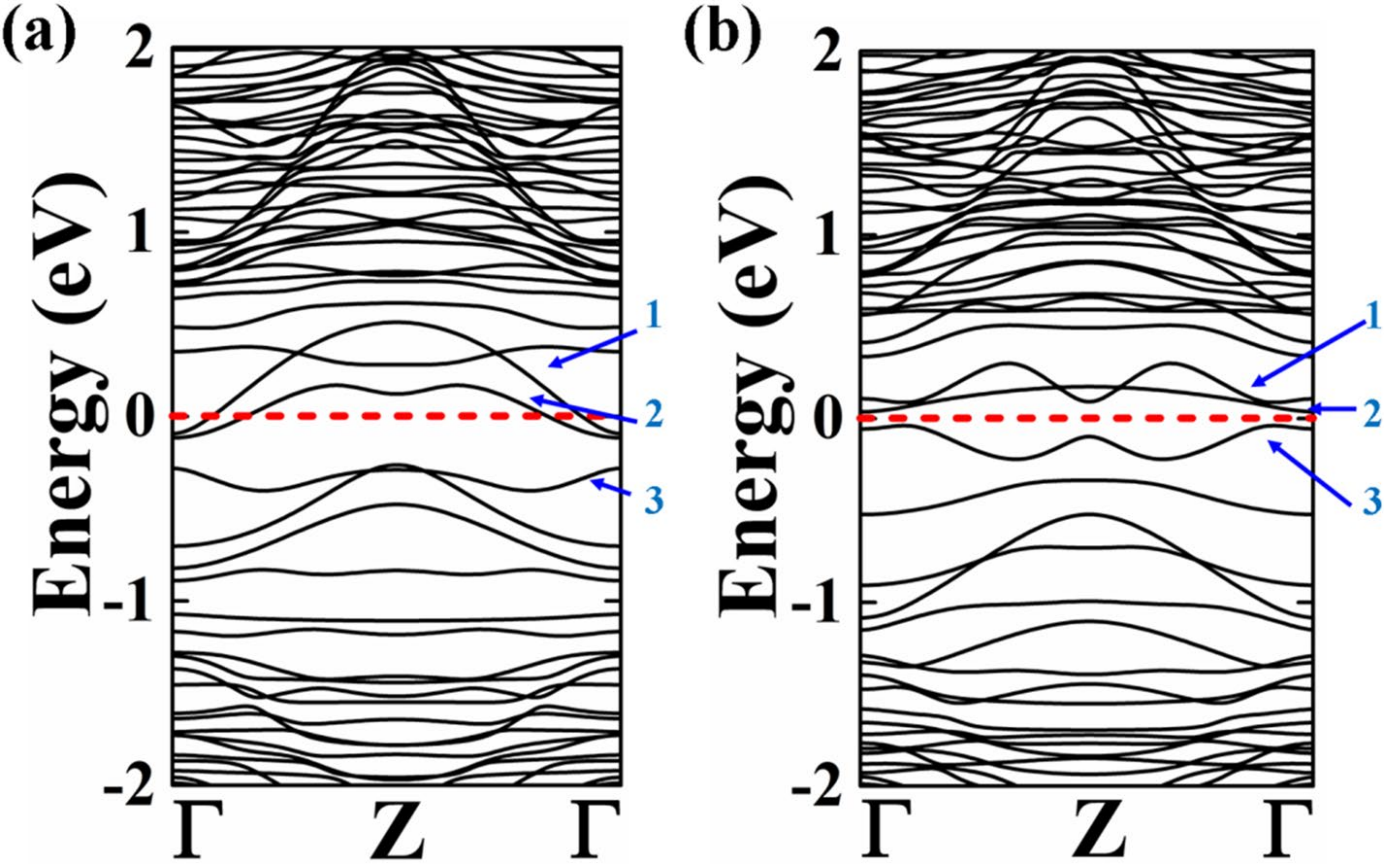
Electronic band structure of (**a**) NW with the Sb vacancy and (**b**) NW with the Ge vacancy.

**Figure 4:**
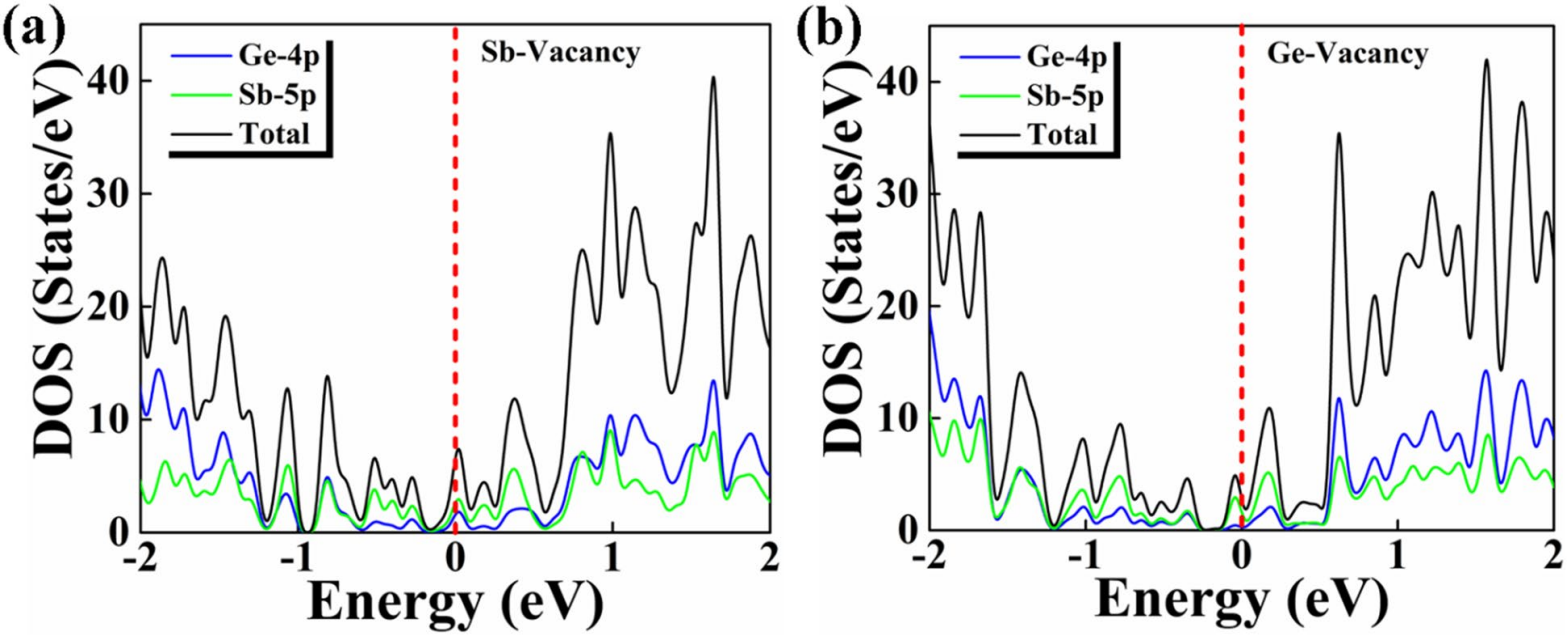
Partial density of states of the Sb/Ge NW with (**a**) the Sb vacancy and (**b**) the Ge vacancy.

Next we have investigated the effects of the vacancy on the electronic properties of the Sb/Ge NWs. The electronic band structures are shown in Fig. 3 and the corresponding PDOS in Fig. 4.

The presence of the Sb (Ge) vacancy causes important changes in the electronic properties of the NW. The core/shell nanowire with the Sb vacancy shows a metallic behaviour with still a 2G_o_ conductance, but changes in the band dispersion across the Fermi level occur. We individuate three primary band lines near the Fermi level. In the pristine Sb/Ge NWs (Fig. 1b) the band labelled 1 is in the conduction band and crosses the Fermi energy near the Z-point at the zone border, with an almost linear dispersion, and at the Γ-point. Another band in the valence crosses the first band at the Fermi Level near the Γ-point. Four bands meet below the Fermi level at the Z-point. In the case of the Sb/Ge NW with the Sb vacancy, instead, the bands 1 and 2 in the conduction cross the Fermi Level only near the Γ-point. Band 3 in the valence does not cross the Fermi Energy. The Sb/Ge NW with the Ge vacancy shows a minor band gap of 0.07 eV between the second and third band line at the Fermi level. The third band is also observed to be shifted upward near the Fermi level.

The inspection of the PDOS reveals that now the density of states of the defective NWs show peaks and gaps near the Fermi Level making the DOS higher and less symmetric on the two sides of the Fermi Level. Also in the NWs with the vacancies the Sb orbitals contribute more in the energy region around the Fermi Level. We can notice a difference between the two density of states corresponding to the Sb and the Ge vacancies: the DOS of the NW with the Sb vacancy is in correspondence of a peak while that of the NW with the Ge vacancy corresponds to a minimum (in reality zero but the used broadening has cancelled in Fig. 4 the small gap). This difference has, as we will see below, a large effect on the thermoelectric figure of merit.

### Thermoelectric properties

In this section, we present the thermoelectric properties of the Sb/Ge NWs with and without the vacancy. We have considered that the lead and the scattering region are made of the same nanowire material. The scattering region contains three unit cells with a total length of 12.5 Å, sufficiently long to avoid leakage current^43^.

**Figure 5:**
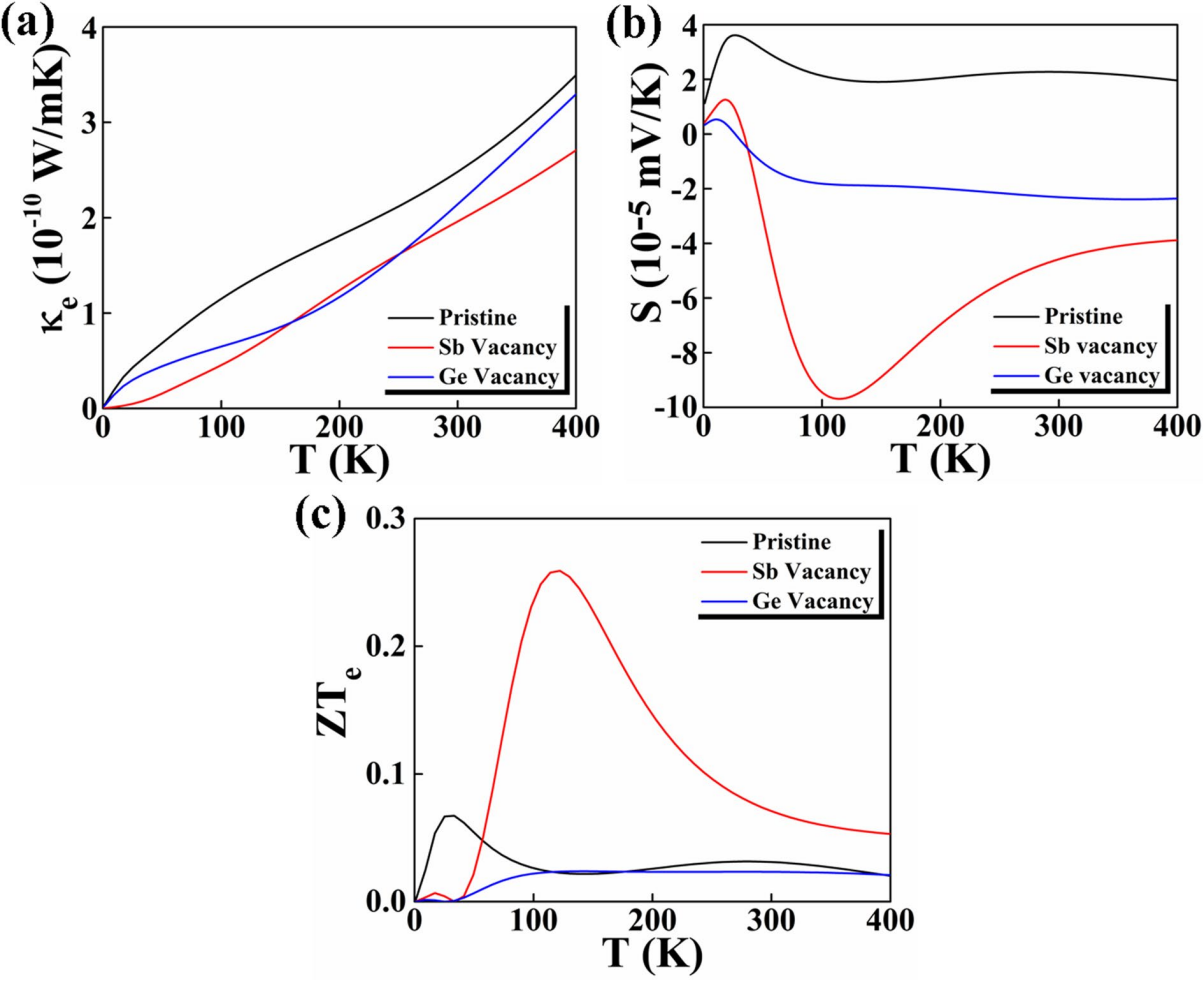
(**a**) Calculated electronic thermal conductivity, (**b**) Seebeck co-efficient and (**c**) figure of merit (ZT_e_) of the pristine Sb/Ge NW, the Sb/Ge NW with the Sb vacancy and the Sb/Ge NW with the Ge vacancy as functions of temperature.

We have calculated the electronic contribution to thermal conductance ($\kappa_{e}$), Seebeck coefficient (S) and figure of merit (ZT_e_) of the nanowires to analyze their energy conversion efficiency (Fig. 5). The ZT_e_ for the NWs is obtained by neglecting the phononic contribution ($\kappa_{p}$) to the thermal conductivity in Eq. (1). Hatef Sadeghi et al. have explained that nanostructures exhibit significantly lower phonon thermal conductances $\kappa_{p}$. Although acoustic phonons are the main heat carriers in bulk crystals, but while reducing the dimensionality, the phonon boundary scattering of the material increases and phononic contribution to thermal conductivity significantly decreases. This leads to change in the phonon density of states and modified phonon dispersion in low-dimensional materials. Therefore, this reasonable approximation to the full figure of merit ZT provided by the electronic thermal conductance ($\kappa_{e}$) leads to higher value^14,19,44^. Moreover, in our manuscript we are comparing ZT_e_ for similar nanowires thus the lattice conductance has to be similar in these systems. Equation 1 shows that the material should have low $\kappa_{e}$ values and high S values to be considered for thermoelectric applications. The pristine Sb/Ge NW shows a thermal conductance of 2.48 × 10^–10^ W/mK at 300 K. The obtained S value at 300 K is 2.28 × 10^–5^ mV/K. Experimentally, it is shown that the core/shell Bi/Te NW has a Seebeck coefficient of 10^–4^ mV/K order of magnitude^34^. The low S value obtained for the Sb/Ge NW lead to a low ZT_e_. The highest figure of merit in the pristine NW is 0.07 at 30 K, which indicates a low efficiency. Interestingly, we obtain an increase in the ZT_e_ value with the presence of the Sb vacancy. The $\kappa_{e}$ value decreases to 1.98 × 10^–10^ W/mK at 300 K (2.15 × 10^–10^ W/mK at 300 K) when a Sb (Ge) vacancy is present. The Seebeck coefficient value instead increases to 2.31 × 10^–5^ mV/K and 4.59 × 10^–5^ mV/K at 300 K for the Sb/Ge NW with Ge and Sb vacancy, respectively. The Seebeck co-efficient values are negative for the NWs with the vacancies because in these cases the carriers are negatively charges, whereas in the pristine NW the carriers are positively charged. These changes in the $\kappa_{e}$ and S values lead to changes in ZT_e_. The highest ZT_e_ is 0.26 calculated for the Sb/Ge NW with the Sb vacancy at 120 K. The enhancement of ZT_e_ with the Sb vacancy formation is related to the presence of sharp features in the electronic density of states at the Fermi Level (Fig. 4)^19^.

Here we consider only the electronic contribution to the figure of merit. However, it has been shown that the presence of point defects in a material increases the phonon scattering and reduces the phonon contribution to the thermal conductance. Thus, the increase in the Figure of Merit we obtain for the defective NWs considering only the electronic contribution is actually underestimated^45^.

**Figure 6:**
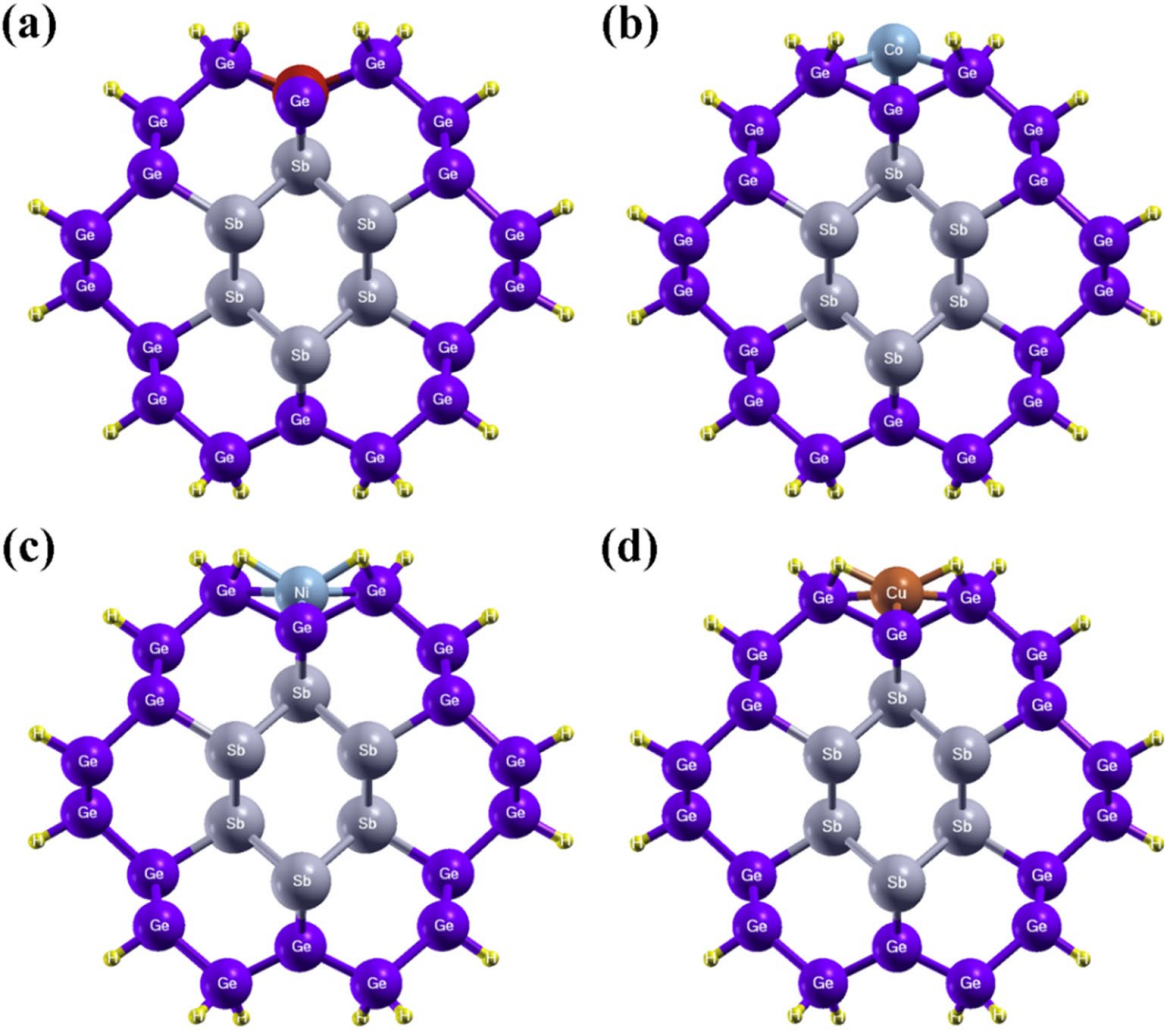
Optimized atomic structure of (**a**) Fe, (**b**) Co, (**c**) Ni and (**d**) Cu doped Sb/Ge NWs in the presence of the Sb vacancy in the core.

We have then considered a further modification of the NW with the Sb vacancy in the core by doping it with transition metals, such as Fe, Co, Ni and Cu, which have been added to the shell as shown in Fig. 6. For these calculations the spin–orbit coupling on the transition metals has been added to the Hamiltonian. After the structural optimization, the Fe and Ni atoms form bonds with both Ge and Sb, while Co and Cu form bonds only with Ge. The bond length between Fe and Ge is 2.25 Å and between Fe and Sb is 2.38 Å; while, Ni forms bonds with Ge and Sb with a bond lengths of 2.35 Å and 2.78 Å, respectively. The bond lengths Co-Ge and Cu-Ge are about 2.30 Å and 2.45 Å, respectively. We have also calculated the adsorption energies using the following formula;

10 $$ E_{ads} = E_{Tot(NW + X)} - E_{Tot(NW)} - E_{Tot(X)} $$

where, X denotes the doped transition metal. The Fe, Co, Ni and Cu doped Sb/Ge-NWs shows adsorption values of − 5.53 eV, − 3.5 eV, − 5.14 eV and − 1.98 eV, respectively. The negative value of adsorption energy indicates that transition metals are energetically favorable for adsorption on Sb/Ge-NWs.

**Figure 7:**
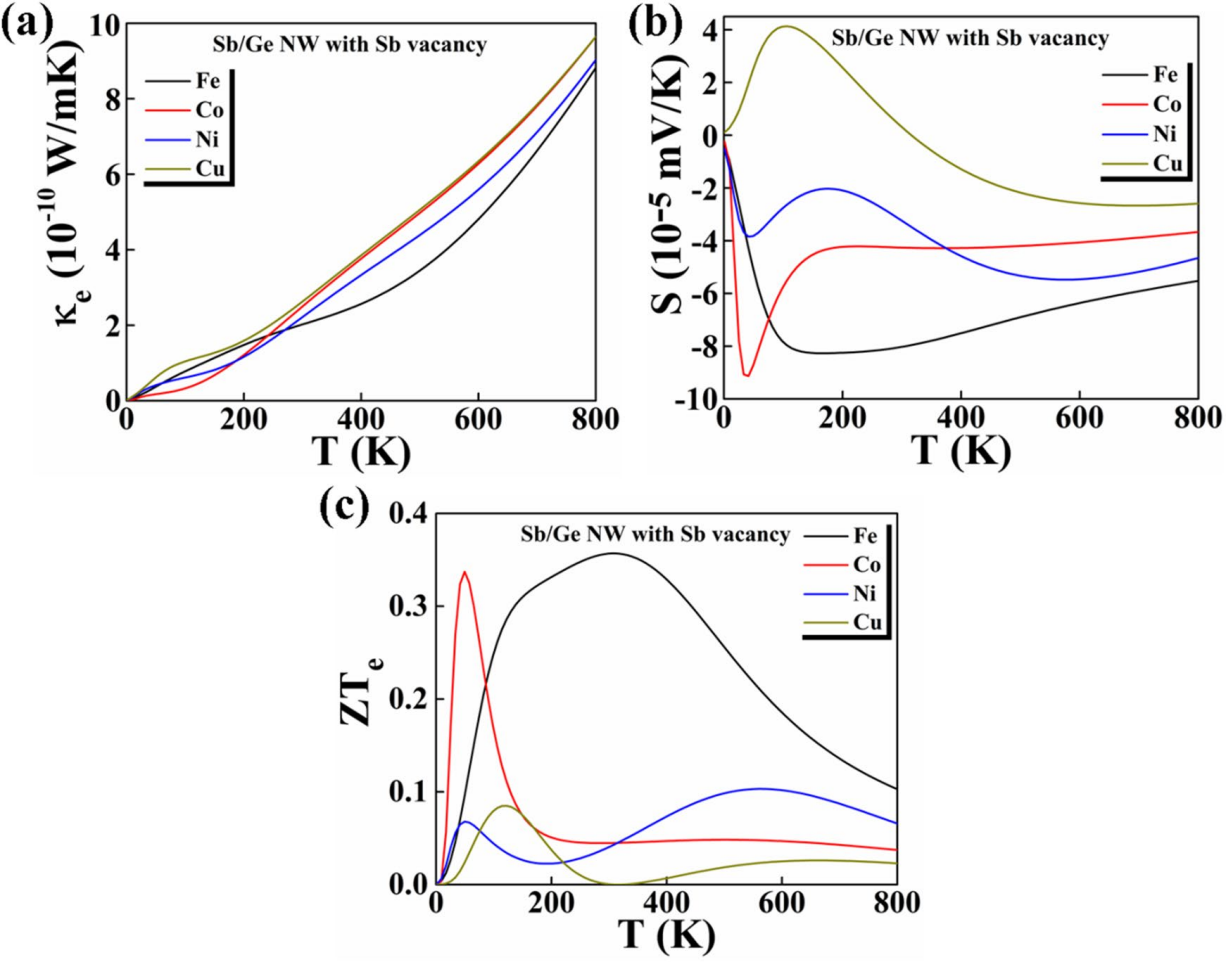
(**a**) Calculated electronic thermal conductivity, (**b**) Seebeck co-efficient and (**c**) figure of merit (ZT_e_) of Sb/Ge NWs with Sb vacancy and one Fe, Co, Ni and Cu atom in the shell as a function of temperature.

We observe that the Fe doped Sb/Ge nanowire shows a lower $\kappa_{e}$ value, 2.01 × 10^–10^ W/mK at 300 K, compared to the Co, Ni and Cu doped NWs (Fig. 7a). In case of Co, Ni and Cu doping, the NWs show $\kappa_{e}$ values of 2.46 × 10^–10^ W/mK, 2.22 × 10^–10^ W/mK and 2.62 × 10^–10^ W/mK at 300 K, respectively. Further, we obtain a higher Seebeck coefficient value in the case of Fe (and Co) doping (as shown in Fig. 7b). The Fe-, Co and Ni-doped Sb/Ge NWs show negative S values, while the Cu doped shows a positive S value of only 2.95 × 10^–5^ mV/K at 300 K. For Fe-, Co and Ni-doped NWs, the S values are 8.05 × 10^–5^ mV/K, 4.25 × 10^–5^ mV/K and 3.24 × 10^–5^ mV/K at 300 K, respectively. The highest ZT_e_ values for the Ni- and Cu-doped Sb/Ge NWs are around 0.1 at 563 K and 0.08 at 120 K, respectively (Fig. 7c). A high ZT_e_ value of 0.33 is obtained for Co doping, but only at the low temperature of 50 K. The highest ZT_e_ value is obtained for Fe doping, 0.36, and this large figure of merit is maintained over a large range of temperatures around 305 K, which is near the room temperature. Thus, creating defects such a vacancy in the core and adding additional atoms in the shell we are able to increment the ZT_e_ of the Sb/Ge NWs up to 80%.

**Figure 8:**
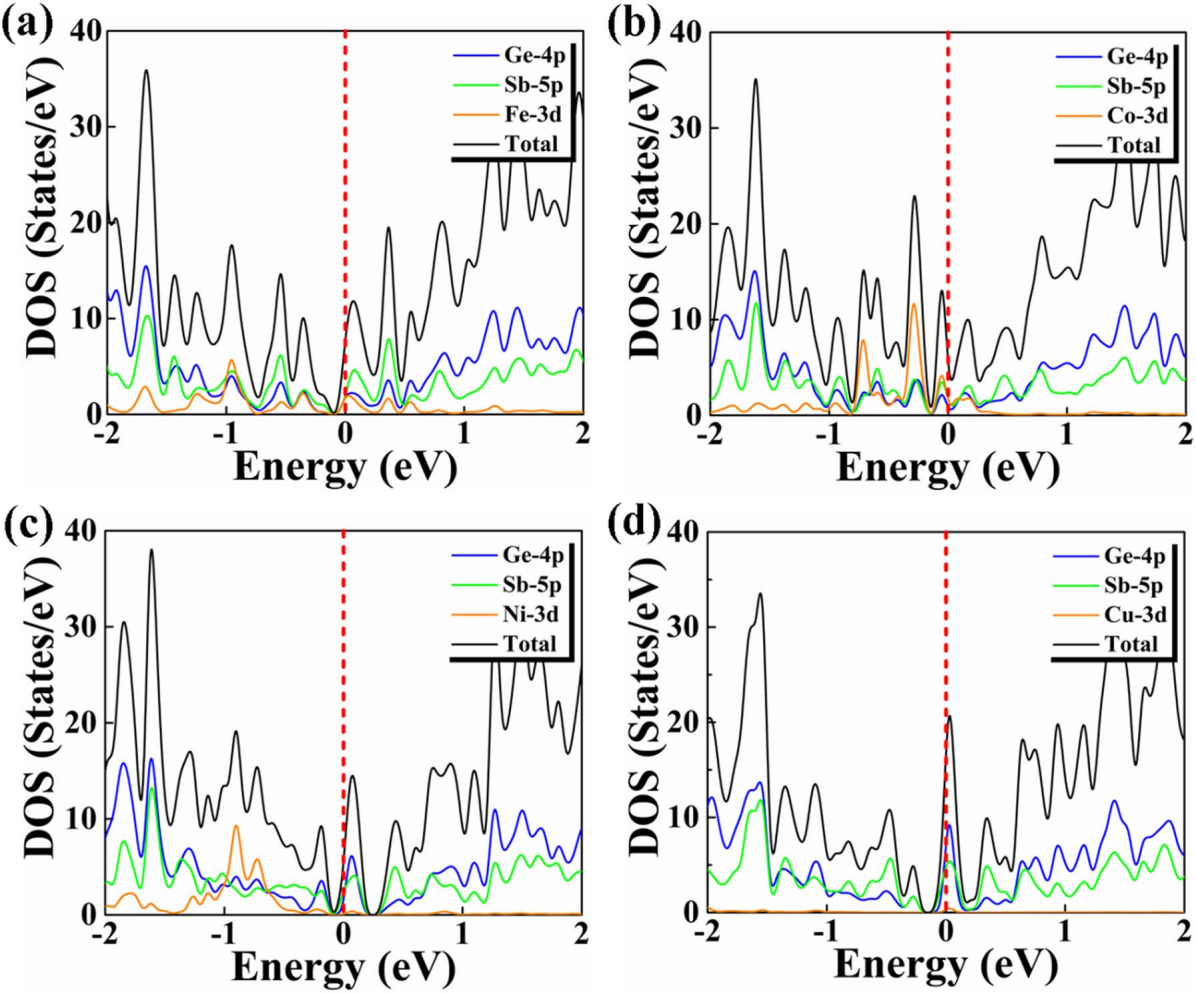
The partial density of states graph of (**a**) Fe, (**b**) Co, (**c**) Ni and (**d**) Cu doped Sb/Ge NW with the Sb vacancy.

The increase in ZT_e_ value can be explained from the analysis of the density of states (Fig. 8). There are sharp features due to Fe and Co at the Fermi level for Fe- and Co-doped NWs, which enhance the thermoelectric efficiency of these NWs^19^. Instead the contributions to the DOS of the Ni and Cu atoms are negligible at the Fermi level, so the change in the ZT_e_ value of these NWs is minor. Moreover in the case of Fe doping the asymmetry of the DOS around the Fermi Level is more pronounced.

## Conclusions

In the present work, we have studied the electronic and thermoelectric properties of Sb/Ge core/shell nanowires by employing the density functional theory. The thermodynamic stability of the nanowire structures is assessed by the calculation of their formation energy. The electronic properties show that the metallic behaviour of the nanowire is due mainly to the Sb-5*p* orbital contribution. The NW with one Sb vacancy in the core exhibits a metallic behaviour with 2G_o_ quantum conductance; while the nanowire with the Ge vacancy shows a minor band gap of 0.07 eV. The study of the thermoelectric properties reveals that the electronic figure of merit of the pristine Sb/Ge NWs is very low, 0.07; however, the efficiency can be increased up to a ZT_e_ value of 0.26 by a vacancy formation at the Sb site. The additional doping with a Fe atom allows the figure of merit to reach the value of 0.36 at ambient temperature. Thus, modifying the atomic structure of the nanowires via vacancy formation and addition of transition metal atoms it is possible to increase the electronic figure of merit of up to 80%.
